# Cross-Cultural Adaptation and Preliminary Psychometric Evaluation of the Malay Orthodontic Patient Satisfaction Questionnaire and Patient Satisfaction Following Fixed Orthodontic Treatment in Malaysia

**DOI:** 10.3390/healthcare14172752

**Published:** 2026-08-28

**Authors:** Al Imran Shahrul, Yap Jia Qi, Shawn Tan Jun Xue, Rohaya Megat Abdul Wahab, Alizae Marny Fadzlin Syed Mohamed

**Affiliations:** Department of Family Oral Health, Faculty of Dentistry, Universiti Kebangsaan Malaysia, Kuala Lumpur 50300, Malaysia; a188197@siswa.ukm.edu.my (J.Q.Y.); a185706@siswa.ukm.edu.my (S.J.X.T.); rohaya_megat@ukm.edu.my (R.M.A.W.)

**Keywords:** orthodontic treatment, patient satisfaction, questionnaire validation, cross-cultural adaptation, Malaysia

## Abstract

**Background:** Orthodontic treatment is a long-term intervention in which patient satisfaction is an important outcome alongside clinical measures. This study aimed to translate and culturally adapt the Orthodontic Patient Satisfaction Questionnaire (OPSQ) into Malay (M-OPSQ), evaluate its content validity, face validity, internal consistency and structural validity, and assess satisfaction following fixed appliance treatment. **Methods:** Content validity was evaluated by eight orthodontists using the Content Validity Index and Content Validity Ratio. Translation involved forward translation, cognitive debriefing, back translation and review by the original questionnaire developer. Face validity was assessed among 34 participants. Preliminary psychometric evaluation involved 159 adults recruited using convenience and snowball sampling. Internal consistency was assessed using Cronbach’s alpha, while structural validity was evaluated using exploratory and confirmatory factor analyses. Exploratory group comparisons were analysed using non-parametric tests. **Results:** Content and face validity assessments resulted in a final 31-item M-OPSQ. Participants had a mean age of 27.11 ± 8.02 years, and 72.3% were female. The mean total score was 159.18 ± 20.33, with excellent internal consistency (α = 0.954; 95% confidence interval (CI) 0.942–0.963). Exploratory factor analysis supported a three-factor structure. The three-factor confirmatory model showed better relative fit than the unidimensional model, although most fit indices remained outside acceptable thresholds. Exploratory analyses identified differences across treatment provider and setting groups. **Conclusions:** The M-OPSQ demonstrated acceptable content and face validity and excellent internal consistency among Malaysian adults who had completed fixed appliance orthodontic treatment. Independent confirmation of the proposed factor structure, together with further assessment of construct validity and test–retest reliability, is required.

## 1. Background

Orthodontic treatment is a complex healthcare intervention that involves prolonged interaction between clinicians and patients, often extending over several years [1,2]. Given the long duration of treatment and the commitment required from both patients and clinicians, patient satisfaction represents an important indicator of treatment success beyond objective clinical outcomes [3]. Satisfaction following orthodontic treatment is influenced by multiple factors, including dentofacial aesthetics, functional improvement, psychosocial benefits, and the quality of interactions between patients and dental professionals [4,5]. Healthcare providers and institutions therefore have an obligation to evaluate patient satisfaction as part of quality assurance processes, enabling continuous improvement of clinical services and patient-centred care.

Malaysia is a multilingual country in which Malay serves as the national language and is widely used across healthcare settings. Although several instruments have been developed to assess satisfaction with orthodontic treatment [6,7,8], few have been formally translated and validated for the Malaysian population. For a questionnaire to be appropriately applied within a specific population, translation alone is insufficient, and formal cross-cultural adaptation and validation are essential to ensure linguistic accuracy, conceptual equivalence, and measurement reliability [9]. Furthermore, the availability of a validated local-language instrument enables benchmarking of patient satisfaction outcomes against international standards and facilitates comparison of research across different populations.

Several instruments have been developed to assess patient-reported outcomes following orthodontic treatment. The post-treatment questionnaire developed by Yassir et al. (2017) [7] provides a concise assessment of perceived changes following treatment, including dental and facial appearance, self-confidence, social interaction, occlusion, chewing and oral hygiene. However, it focuses primarily on perceived treatment-related changes rather than the broader experience of patient satisfaction.

The Orthodontic Patient Satisfaction Questionnaire (OPSQ) was developed as a 58-item instrument encompassing six domains: doctor–patient relationship, situational aspects of treatment, dentofacial improvement, psychosocial improvement, dental function, and a residual category [10]. Compared with the Yassir post-treatment questionnaire, the OPSQ evaluates a broader range of satisfaction-related experiences, including clinician–patient interaction and situational aspects of treatment alongside perceived dentofacial, psychosocial and functional outcomes.

The OPSQ has subsequently undergone linguistic and cross-cultural adaptation in several populations, including Brazilian Portuguese [11], Arabic [12], Malay [13], and United Kingdom (UK) populations. The UK adaptation reduced the original 58-item instrument to 37 items through expert panel review and removal of items with low item–total correlations [14]. Factor analysis did not support the original six-domain structure; consequently, the subscales were removed, and the 37-item instrument was scored as a single measure of overall orthodontic patient satisfaction.

The present study therefore adapted the 37-item UK version rather than the original 58-item OPSQ because it had already undergone item-level psychometric evaluation and its shorter length reduced respondent burden. Furthermore, an appropriately cross-culturally adapted and evaluated Malay-language version of the reduced instrument was not available. Therefore, the objective of this study was to translate and culturally adapt the OPSQ into Malay, evaluate its content validity, face validity, internal consistency and structural validity, and assess patient satisfaction following treatment with pre-adjusted edgewise fixed appliances in a Malaysian population.

## 2. Methodology

### 2.1. Ethical Approval

Ethical approval was obtained from the Research Ethics Committee of the Universiti Kebangsaan Malaysia (UKM) (Reference: JEP-2024-809).

### 2.2. Content Validity

The content validity of the UK 37-item version of the OPSQ [14] was evaluated by an expert panel comprising eight orthodontists. The panel evaluated each item for its relevance and essentiality for use among patients aged 18 years and older in the Malaysian population [15]. Content validity was assessed using the item-level Content Validity Index (I-CVI), universal agreement (UA), and the Content Validity Ratio (CVR) [16].

For the I-CVI assessment, each item was rated on a four-point scale: 1 = not relevant, 2 = needs major revision, 3 = needs minor revision, and 4 = very relevant. Ratings of 1 and 2 were classified as irrelevant, whereas ratings of 3 and 4 were classified as relevant [17,18]. The I-CVI was calculated by dividing the number of experts who rated an item as 3 or 4 by the total number of experts. I-CVI values ranged from 0 to 1. Accordingly, items rated as relevant by at least seven of the eight experts were considered acceptable (I-CVI ≥ 0.875), items rated as relevant by six experts required further consideration (I-CVI = 0.75), and items with agreement from five or fewer experts (I-CVI ≤ 0.625) were considered for elimination.

The UA was used to assess the level of complete agreement among experts regarding the relevance of items, based on the I-CVI ratings. A UA score of 1 was assigned when all experts rated an item as relevant, meaning each expert assigned a score of 3 or 4. Conversely, a score of 0 was assigned when there was a lack of complete agreement among the experts [16].

Item essentiality was evaluated separately using the CVR [15,19]. Experts rated each item on a four-point scale: 1 = not necessary, 2 = useful but not essential, 3 = may be essential, and 4 = definitely essential. Ratings of 1 and 2 were categorised as “not essential”, while ratings of 3 and 4 were categorised as “essential”. The CVR was calculated using the formula CVR = (ne − N/2)/(N/2), where ne represents the number of experts who rate an item as essential and N represents the total number of experts. CVR values ranged from −1 to +1, with higher values indicating greater agreement among experts regarding item necessity. For a panel of eight experts, the minimum acceptable CVR value was set at 0.75 [19]. Qualitative feedback primarily concerned wording perceived as ambiguous or less natural in Malay. These comments were considered together with the quantitative item-level Face Validity Index (I-FVI) results when determining whether items required modification, retention or deletion.

### 2.3. Translation

The cross-cultural adaptation of the OPSQ into Malay was informed by the International Society for Pharmacoeconomics and Outcomes Research (ISPOR) Principles of Good Practice for the Cross-Cultural Adaptation Process for Patient-Reported Outcome Measures [20], with modifications according to the study procedures and available documentation. Forward translation of the original English questionnaire into Malay was undertaken independently by two professional translators appointed by the Malaysian Institute of Translation and Books. The translators’ specific demographic and professional backgrounds were not systematically documented. Translation focused on conceptual rather than literal equivalence.

The forward-translated versions were reviewed by an orthodontist and a Malay-language specialist for content accuracy, clarity, and conceptual equivalence. Discrepancies were discussed and resolved by consensus. Where clarification or modification of the translation was required, the Malaysian Institute of Translation and Books was consulted, and the necessary amendments were made. The reconciled version formed the first version of the Malay OPSQ (M-OPSQ).

This initial version underwent preliminary cognitive debriefing with five participants, consistent with the sample of five to eight respondents recommended by the ISPOR Principles of Good Practice for the Translation and Cultural Adaptation Process for Patient-Reported Outcomes Measures [20]. Structured cognitive interviews were conducted to assess participants’ understanding of the questionnaire, with particular attention to general phrasing and terms considered potentially ambiguous by the research team. Participant feedback was reviewed and incorporated by consensus, with necessary modifications made to improve clarity and comprehensibility, resulting in the second version of the M-OPSQ.

The second version was subsequently back-translated into English by two further independent linguistic experts from the Malaysian Institute of Translation and Books. The resulting back-translated questionnaire was submitted to the original developer for evaluation. Each item was assessed against three criteria: (1) conceptually and linguistically equivalent to the original item; (2) functionally equivalent with grammatical differences; and (3) lack of equivalence being evident [21]. Incorporating the developer feedback, the research group implemented refinements to the questionnaire, producing Version 3 of the M-OPSQ.

### 2.4. Face Validity

The comprehension of the Version 3 M-OPSQ was then face validated by a panel comprising 34 participants, including 6 orthodontists, 7 orthodontic postgraduate students, and 21 native Malay-speaking patients representing the target population [22].

Face validity was assessed quantitatively by analysing respondents’ ratings of the clarity and comprehensibility of each item within the various domains. Each item was rated on a four-point scale: 1 = not clear or understandable, 2 = somewhat clear or understandable, 3 = clear and understandable, and 4 = very clear and understandable. Ratings of 1 and 2 were considered to indicate inadequate clarity or comprehensibility, whereas ratings of 3 and 4 were considered to indicate acceptable clarity and comprehensibility.

The I-FVI was calculated by dividing the number of respondents who rated an item as 3 or 4 by the total number of respondents. I-FVI values ranged from 0 to 1, with a minimum value of 0.83 indicating acceptable face validity [15,22,23]. This cut-off was adopted from previously published face validity studies using comparable rater panels and represents a conventional threshold.

### 2.5. M-OPSQ Final Version

Following the face and content validity assessments, the research team reviewed the corresponding ratings and item-level feedback. Each item was further reviewed by an orthodontist and a Malay-language expert to determine whether modification, retention, or removal was required. Additional consultation with the Malaysian Institute of Translation and Books or the original questionnaire developer was available if clarification was required, but this was not necessary. Based on this integrated review, Version 4 of the M-OPSQ was finalised.

### 2.6. Psychometric Evaluation

#### 2.6.1. Data Collection

A cross-sectional study design was employed. Participants were recruited using convenience and snowball sampling. Eligible individuals were briefed on the study objectives, and written informed consent was obtained before administration of the M-OPSQ. Data were collected using Google Forms. Responses were treated as confidential rather than anonymous because email addresses and telephone numbers were recorded solely to identify and prevent duplicate submissions. These identifiers were not included in the statistical analyses. Questionnaire items were presented in a fixed order to all participants, all fields were mandatory, and incomplete questionnaires therefore could not be submitted.

#### 2.6.2. Sample Size

The sample size was guided by a commonly used ratio of five participants per questionnaire item [24]. With 31 items in the M-OPSQ, a minimum of 155 participants was required. This rule of thumb was considered appropriate for preliminary scale evaluation and assessment of internal consistency but was not intended to establish adequacy for comprehensive psychometric validation. No separate a priori power calculation was performed for the exploratory secondary between-group comparisons.

#### 2.6.3. Study Population

Participants from the Faculty of Dentistry UKM were identified from the patient registry of individuals who had completed orthodontic treatment. In addition, the questionnaire Google link was distributed and shared via WhatsApp to dental colleagues and personal contacts, including individuals who had completed orthodontic treatment outside UKM. Snowball recruitment was encouraged, allowing recipients to share the questionnaire link with other eligible participants.

The study population comprised individuals aged 18 years and older who had completed orthodontic treatment and were proficient in the Malay language. Individuals with complex medical histories or learning disabilities that could impair comprehension of the questionnaire were excluded from the study. The interval between completion of orthodontic treatment and questionnaire administration was not recorded and may therefore have varied from recent treatment completion to several years after treatment.

### 2.7. Statistical Analysis

Microsoft Excel was used for data entry and tabulation of the I-CVI, CVR and I-FVI. Statistical analyses were conducted using IBM SPSS Statistics version 31. Descriptive statistics were used to summarise participant characteristics and item responses. Item and total M-OPSQ scores were summarised using means and standard deviations. Internal consistency was assessed using Cronbach’s alpha (α), with the corresponding 95% confidence interval reported.

Structural validity was assessed using exploratory factor analysis (EFA) and confirmatory factor analysis (CFA). Sampling adequacy was evaluated using the Kaiser–Meyer–Olkin (KMO) measure and Bartlett’s test of sphericity. EFA was conducted using principal axis factoring with Direct Oblimin rotation. Factor retention was determined primarily by parallel analysis based on 1000 simulated datasets, supported by scree plot inspection and eigenvalues.

Maximum likelihood CFA was used to compare a unidimensional model reflecting the UK source instrument with the factor structure identified by the EFA. Model fit was assessed using χ^2^/df, Comparative Fit Index (CFI), Tucker–Lewis Index (TLI), Root Mean Square Error of Approximation (RMSEA) and Standardised Root Mean Square Residual (SRMR). Values of χ^2^/df ≤ 3.0, CFI and TLI ≥ 0.90, and RMSEA and SRMR ≤ 0.08 were considered indicative of acceptable fit. Structural validity analyses were based on complete cases. As the EFA and CFA were conducted using the same sample, the CFA was interpreted as a preliminary model comparison rather than an independent confirmation of the factor structure.

Exploratory secondary analyses compared total M-OPSQ scores across selected participant and treatment characteristics. Total scores were summarised using medians and interquartile ranges (IQRs). Gender differences were assessed using the Mann–Whitney U test, whereas treatment provider and treatment setting were compared using the Kruskal–Wallis test. Significant Kruskal–Wallis results were followed by pairwise Mann–Whitney U tests using a Bonferroni-adjusted significance threshold of α = 0.0083. Effect sizes were reported for all between-group comparisons to complement statistical significance. Rank-based r was interpreted using the dental research thresholds proposed for correlation-based effect sizes as negligible (<0.20), small (0.20–0.39), medium (0.40–0.69) or large (≥0.70) [25]. Eta squared (η^2^H) was interpreted as negligible (<0.01), small (0.01–0.05), medium (0.06–0.13) or large (≥0.14) [26]. Between-group differences and corresponding 95% confidence intervals were estimated using the Hodges–Lehmann method [27].

## 3. Results

### 3.1. Content Validity and Face Validity

Content and face validity results of the M-OPSQ are presented in Table 1. I-CVI values ranged from 0.75 to 1.00. Items 13 and 29 had I-CVI values of 0.75, corresponding to agreement by six of the eight experts and therefore requiring further consideration. All other items were rated as relevant by at least seven of the eight experts, corresponding to I-CVI values of ≥0.875. Most items met the minimum CVR threshold of 0.75; however, Items 20, 29, 32 and 37 did not meet this criterion. Universal agreement was achieved for most items, reflecting a high level of agreement among the expert panel regarding item relevance.

Face validity assessment demonstrated generally good clarity and comprehensibility. Most items met the prespecified I-FVI threshold of 0.83, while five items (Items 4, 5, 6, 13 and 20) had I-FVI values below this threshold. Following an integrated review of content validity, face validity and qualitative feedback, Items 4, 13 and 20 were removed. Items 5 and 6 were retained because their content validity findings supported their relevance despite lower face validity scores. Items 29, 32 and 37 were removed primarily on the basis of their content validity findings. Overall, 31 items were retained, resulting in the final Version 4 of the M-OPSQ.

### 3.2. Psychometric Evaluation

A total of 159 participants were included. The mean age was 27.11 ± 8.02 years, with females comprising 72.3% of the sample. Most participants reported receiving orthodontic treatment from orthodontic postgraduate students (64.2%), followed by orthodontic specialists (22.0%), general dentists (8.2%) and those who were unsure of their treatment provider (5.7%). Most participants had received treatment at the Faculty of Dentistry UKM (75.5%), followed by private dental clinics (17.6%), other university dental faculties (3.8%) and Ministry of Health dental clinics (3.1%). Participant characteristics are presented in Table 2.

Item-level descriptive statistics and internal consistency are presented in Table 3. The mean total M-OPSQ score was 159.18 ± 20.33, while individual item means ranged from 4.53 ± 1.35 to 5.53 ± 0.75. The highest mean scores were observed for items relating to respectful treatment by the orthodontist and orthodontic team, whereas lower mean scores were observed for items relating to perceived educational or career benefits. The 31-item scale demonstrated excellent internal consistency, with a Cronbach’s alpha of 0.954 (95% CI 0.942–0.963).

The distribution of item responses is presented in Table 4. Most items had a high proportion of responses within scores 5–6. The highest proportions were observed for Item 1 (“my teeth are straighter”) and Item 36 (“the orthodontist treated me with respect”), both at 90.6%. The highest proportions of responses within scores 1–2 were observed for Item 12 (“school performance improved”) at 10.1% and Item 25 (“rarely kept waiting for appointments”) at 9.4%.

### 3.3. Structural Validity

Structural validity analyses were based on 158 complete cases. Sampling adequacy was excellent (KMO = 0.919), and Bartlett’s test of sphericity was statistically significant (χ^2^ = 4165.12, df = 465, *p* < 0.001), supporting the suitability of the data for factor analysis.

Parallel analysis supported a three-factor solution, despite five factors having eigen-values greater than 1.0. The first three factors had initial eigenvalues of 13.808, 3.281 and 1.936, respectively, together accounting for 61.37% of the total variance. Direct Oblimin rotation identified three interpretable dimensions: Aesthetics and Psychosocial Benefits, Doctor–Patient Relationship and Environment, and Oral and Dental Function. Inter-factor correlations ranged from 0.35 to 0.59. Pattern coefficients are presented in Table 5. Six items showed secondary pattern coefficients of ≥0.30, although most demonstrated a clear primary loading. Item 12 showed cross-loading on Aesthetics and Psychosocial Benefits (0.399) and Oral and Dental Function (0.374) and was provisionally assigned to the former based on its slightly higher pattern coefficient.

CFA demonstrated poor fit for the unidimensional model (χ^2^/df = 4.99, CFI = 0.570, TLI = 0.539, RMSEA = 0.159, SRMR = 0.117). The three-factor model demonstrated substantially better relative fit (χ^2^/df = 2.74, CFI = 0.813, TLI = 0.799, RMSEA = 0.105, SRMR = 0.097). However, with the exception of χ^2^/df, the fit indices remained outside the predefined criteria for acceptable model fit. Full model fit statistics are presented in Table 6.

### 3.4. Exploratory Comparisons

Exploratory comparisons of total M-OPSQ scores are presented in Table 7. No statistically significant difference was observed between male and female participants (*p* = 0.314). Significant differences were observed across treatment provider and treatment setting groups (both *p* < 0.001).

Participants treated by orthodontic postgraduate students had the highest median M-OPSQ score (167.00 [IQR 23.00]), followed by those treated by orthodontic specialists (152.00 [IQR 27.00]) and general dentists (144.00 [IQR 28.50]). Participants treated at the Faculty of Dentistry UKM had a median score of 165.00 (IQR 22.00), compared with 145.00 (IQR 24.25) among those treated in private dental clinics.

Bonferroni-adjusted post hoc comparisons are presented in Table 8 and Table 9. Significant differences were observed between general dentists and orthodontic postgraduate students (*p* = 0.003) and between orthodontic specialists and orthodontic postgraduate students (*p* = 0.008). For treatment setting, a significant difference was observed between the Faculty of Dentistry UKM and private dental clinics (*p* < 0.001).

## 4. Discussion

### 4.1. Summary of the Results

The present study resulted in a 31-item M-OPSQ following translation, cross-cultural adaptation, and assessment of content and face validity. Preliminary psychometric evaluation in 159 participants demonstrated excellent internal consistency. Structural validity analysis provided preliminary evidence of a multidimensional structure, with EFA supporting three related dimensions. Although the three-factor CFA model demonstrated better relative fit than the unidimensional model, most fit indices remained outside the predefined criteria for acceptable model fit.

Overall post-treatment satisfaction scores were generally high. The highest satisfaction scores were observed for perceived improvement in tooth alignment and respectful treatment by the orthodontic team, whereas lower scores were observed for perceived educational benefits. The study population was predominantly female, with most participants having received treatment at a university dental faculty from orthodontic postgraduate students.

No significant difference in total M-OPSQ scores was observed between genders. Exploratory secondary analyses identified differences in satisfaction scores across treatment provider and treatment setting groups, with higher scores observed among participants treated by orthodontic postgraduate students and those treated at the UKM Dental Faculty. The omnibus effect sizes for treatment provider and treatment setting were of medium magnitude, whereas effect sizes for individual pairwise comparisons were negligible to small [25]. These findings indicated that, although statistically significant group differences were identified, their magnitude varied according to the level of comparison and should be interpreted cautiously.

### 4.2. Interpretation and Comparison to Previous Studies

The M-OPSQ encompassed multiple dimensions of orthodontic treatment experience and perceived outcomes. Several items demonstrated lower content validity, particularly those addressing treatment inconvenience, expectations of aesthetic improvement, psychosocial effects, the clinical environment, and rapport with the clinician. These findings may indicate that some items were perceived by the expert panel as less relevant or less essential for evaluating post-treatment satisfaction in the Malaysian context. However, the reasons for these lower ratings were not formally investigated and should therefore be interpreted cautiously.

Cross-cultural adaptations of the OPSQ have resulted in different numbers of retained items across populations. The Brazilian Portuguese version retained all 58 items and demonstrated excellent internal consistency (α = 0.919) and test–retest reliability (intraclass correlation coesfficient, ICC = 0.918) [11]. The Arabic version retained 39 items with an internal consistency of α = 0.79 [12], while a previous Malay version comprised 42 items and reported an overall Cronbach’s alpha of 0.94 [13]. The UK version used as the source instrument in the present study had been reduced to 37 items and demonstrated high internal consistency (α = 0.92) but weak test–retest agreement [14].

In the present study, six items were removed from the 37-item UK version, resulting in a 31-item M-OPSQ with excellent internal consistency (α = 0.954). However, high internal consistency does not establish unidimensionality and may also reflect overlap among items. Structural validity was therefore further examined using EFA and CFA.

Structural validity analysis suggested that the 31-item M-OPSQ may not be adequately represented by a strictly unidimensional structure. The unidimensional CFA model showed poor fit, whereas EFA supported three related dimensions encompassing Aesthetics and Psychosocial Benefits, Doctor–Patient Relationship and Environment, and Oral and Dental Function. The three-factor CFA model demonstrated substantially better relative fit than the unidimensional model, although most fit indices remained outside conventional acceptable thresholds. These findings therefore provide preliminary evidence of multidimensionality rather than confirmation of a definitive three-factor structure.

The findings differ from the UK adaptation [14], in which the original six-domain structure was not supported and the questionnaire was subsequently scored as a single overall measure. These findings suggest that the dimensional organisation of orthodontic patient satisfaction may differ across populations following linguistic and cultural adaptation. Interpretation should nonetheless remain cautious, as EFA and CFA were conducted using the same sample, and several items, most notably item 12, demonstrated imperfect factorial separation. Subscale scoring based on the proposed three-factor structure is therefore not recommended at this stage. Replication in a larger independent sample is required to confirm the factor structure, determine whether further item refinement is needed, and assess test–retest reliability.

The present study differed from previous Malaysian work [13] mainly in its documented translation and cross-cultural adaptation process and quantitative assessment of content and face validity. Previous Malay-language studies did not report comparable detail regarding translation procedures or quantitative validity indices [13,28]. Direct comparison should nevertheless be made cautiously because the previous Malay questionnaire retained the original subscales, whereas the present M-OPSQ was scored primarily as an overall measure, while its proposed multidimensional structure was explored separately.

Overall satisfaction scores were generally high, consistent with previous Malaysian and international studies. However, comparisons should be interpreted cautiously because of differences in instruments, populations, sampling methods and treatment settings. Exploratory secondary analyses identified differences across treatment provider and clinical setting groups, but these findings should not be interpreted as evidence of differences in quality of care because important potential confounding factors were not measured or controlled.

### 4.3. Generalisability and Limitations

Malaysia comprises diverse ethnic and cultural groups, and interpretation of some Malay terms may vary across regions or communities. Although standard Malay was used throughout the questionnaire, consistent interpretation across different demographic groups was not specifically evaluated. Convenience and snowball sampling may also have introduced selection bias, while the predominance of participants from a single institution limits generalisability to the wider Malaysian orthodontic population.

The interval between treatment completion and questionnaire administration was not recorded, and recall-based items may therefore have been affected by recall bias. Satisfaction may also have changed over time.

Structural validity findings should be regarded as preliminary because EFA and CFA were conducted using the same modest sample and the three-factor model did not meet most conventional fit criteria. Other aspects of construct validity, including convergent and discriminant validity and test–retest reliability, were not assessed. Criterion validity was also not evaluated because no appropriate gold standard instrument was available.

The evaluation was restricted to adults treated with pre-adjusted edgewise fixed appliances, limiting applicability to younger populations and other treatment modalities. Treatment provider was self-reported, and some participants may have been managed by more than one clinician. Potential confounding factors, including case complexity, treatment characteristics and institutional differences, were not measured or controlled; therefore, provider and setting comparisons should be interpreted as exploratory.

The sample size was appropriate for preliminary psychometric evaluation but not comprehensive validation, and no a priori power calculation was performed for the exploratory secondary comparisons. Finally, although forward and back translations were undertaken independently by professional linguistic experts, detailed information regarding their professional backgrounds and blinding was not systematically recorded; therefore, complete adherence to all recommended ISPOR reporting elements could not be confirmed.

## 5. Conclusions

The M-OPSQ demonstrated acceptable content and face validity and excellent internal consistency following translation and cross-cultural adaptation among Malaysian adults who had completed fixed appliance orthodontic treatment. Structural validity analysis provided preliminary evidence of a multidimensional structure; however, the proposed three-factor model requires confirmation in a larger independent sample. Further assessment of construct validity and test–retest reliability is also required before the M-OPSQ can be considered comprehensively validated. Additional evaluation is needed to determine its applicability to younger age groups and other orthodontic treatment modalities.

## Figures and Tables

**Table 1:** Content and Face Validity Results of the M-OPSQ.

Item No.	Item	Content Validity				Face Validity		Decision
		No. of Panel Agree On The Relevancy of Item	I-CVI	UA	CVR	No. of Panel Agree on the Clarity and Comprehensibility of Item	I-FVI	
1	Now that the orthodontic treatment is complete, my teeth are straighter.	8	1.00	1	0.75	30	0.88	Retained
2	Now that orthodontic treatment is complete, I find it easier to eat.	7	0.88	0	0.75	31	0.91	Retained
3	Since orthodontic treatment, I feel that I am more attractive.	8	1.00	1	0.75	32	0.94	Retained
4	I thought that my appearance would improve more than it did.	7	0.88	0	1	20	0.59	Deleted
5	My appearance has changed as I expected.	8	1.00	1	0.75	28	0.82	Retained
6	The way my teeth come together is better since I have been treated.	8	1.00	1	0.75	27	0.79	Retained
7	I feel satisfied about the way my appearance has changed since finishing orthodontic treatment.	8	1.00	1	1	33	0.97	Retained
8	I generally feel happier about my appearance, since finishing orthodontic treatment.	8	1.00	1	1	31	0.91	Retained
9	I feel happier in myself because I look so much better since I have been treated.	7	0.88	0	1	29	0.85	Retained
10	I generally feel better about myself since my orthodontic treatment.	8	1.00	1	0.75	33	0.97	Retained
11	I believe I can have a better career because of my orthodontic treatment.	7	0.88	0	1	30	0.88	Retained
12	I believe my school performance is better because of orthodontic treatment.	7	0.88	0	1	29	0.85	Retained
13	I am more outgoing because of orthodontic treatment.	6	0.75	0	1	28	0.82	Deleted
14	I generally feel more confident because of my orthodontic treatment.	8	1.00	1	1	33	0.97	Retained
15	Positive comments have been made about my appearance since my orthodontic treatment.	8	1.00	1	1	32	0.94	Retained
16	I find it easier to eat since I have been treated.	7	0.88	0	1	30	0.88	Retained
17	I find it easier to chew since I have been treated.	7	0.88	0	1	29	0.85	Retained
18	I find it easier to bite food since I have been treated.	8	1.00	1	1	31	0.91	Retained
19	I would recommend orthodontic treatment to everyone who has difficulties chewing food.	7	0.88	0	0.75	31	0.91	Retained
20	I found my orthodontic treatment inconvenient.	7	0.88	0	0.25	24	0.71	Deleted
21	I take better care of my teeth since completing orthodontic treatment.	8	1.00	1	0.75	33	0.97	Retained
22	I am satisfied with the results of my orthodontic treatment.	8	1.00	1	1	33	0.97	Retained
23	I was satisfied with the choice of appointment times.	8	1.00	1	1	32	0.94	Retained
24	I was happy with the amount of time the orthodontist spent with me at each appointment.	8	1.00	1	1	33	0.97	Retained
25	I was rarely kept waiting for appointments.	7	0.88	0	1	29	0.85	Retained
26	The waiting area was comfortable.	8	1.00	1	1	34	1.00	Retained
27	The treatment area was clean.	7	0.88	0	1	34	1.00	Retained
28	Problems that occurred with my braces were quickly solved.	8	1.00	1	1	34	1.00	Retained
29	The treatment area was modern and up to date.	6	0.75	0	0.5	33	0.97	Deleted
30	The orthodontist was gentle when treating me.	8	1.00	1	1	32	0.94	Retained
31	Before treatment began, the orthodontist carefully explained my treatment.	8	1.00	1	1	33	0.97	Retained
32	I liked the orthodontist who treated me.	8	1.00	1	0.25	32	0.94	Deleted
33	Questions I had about my treatment were answered promptly.	8	1.00	1	1	34	1.00	Retained
34	The dental nurses were caring when treating me.	8	1.00	1	1	34	1.00	Retained
35	The orthodontic team treated me with respect.	8	1.00	1	1	34	1.00	Retained
36	The orthodontist treated me with respect.	8	1.00	1	1	34	1.00	Retained
37	I liked the orthodontist who treated me.	8	1.00	1	0.5	31	0.91	Deleted

**Table 2:** Participant Demographic Profile and Orthodontic Treatment Characteristics.

Variable	*n* (%)
Age, Mean ± SD	27.11 ± 8.02
Gender	
Male	44 (27.7)
Female	115 (72.3)
Who provided your orthodontic treatment?	
General Dentist	13 (8.2)
Orthodontic Specialist	35 (22.0)
Dental Postgraduate Student	102 (64.2)
Unsure/Do Not Know	9 (5.7)
Where did you receive your orthodontic treatment?	
UKM Dental Faculty	120 (75.5)
Other University Dental Faculty	6 (3.8)
KKM Dental Clinic	5 (3.1)
Private Dental Clinic	28 (17.6)

KKM, Kementerian Kesihatan Malaysia (Ministry of Health Malaysia).

**Table 3:** Item Descriptive Statistics and Internal Consistency of the M-OPSQ.

Item	Mean ± SD	Total Score Mean ± SD	Cronbach’s α (95% CI)
Item 1	5.46 ± 0.74	159.18 ± 20.33	0.954 (0.942–0.963)
Item 2	5.08 ± 1.15		
Item 3	5.41 ± 0.90		
Item 5	4.72 ± 1.13		
Item 6	5.16 ± 0.98		
Item 7	5.22 ± 0.91		
Item 8	5.22 ± 0.91		
Item 9	5.17 ± 0.94		
Item 10	5.30 ± 0.86		
Item 11	4.73 ± 1.18		
Item 12	4.53 ± 1.35		
Item 14	5.27 ± 0.80		
Item 15	4.99 ± 1.01		
Item 16	4.87 ± 1.20		
Item 17	4.82 ± 1.24		
Item 18	4.88 ± 1.17		
Item 19	5.14 ± 1.13		
Item 21	5.10 ± 0.95		
Item 22	5.23 ± 0.98		
Item 23	5.14 ± 0.98		
Item 24	5.22 ± 1.03		
Item 25	4.68 ± 1.39		
Item 26	5.03 ± 1.04		
Item 27	5.44 ± 0.74		
Item 28	5.18 ± 1.08		
Item 30	5.26 ± 0.92		
Item 31	5.23 ± 0.97		
Item 33	5.34 ± 0.90		
Item 34	5.34 ± 0.95		
Item 35	5.51 ± 0.75		
Item 36	5.53 ± 0.75		

**Table 4:** Distribution of Item Responses by Score Category.

Item	Scores 1–2, *n* (%)	Scores 3–4, *n* (%)	Scores 5–6, *n* (%)
Item 1	0 (0.0)	15 (9.4)	144 (90.6)
Item 2	6 (3.8)	35 (22.0)	118 (74.2)
Item 3	3 (1.9)	21 (13.2)	135 (84.9)
Item 5	6 (3.8)	55 (34.6)	98 (61.6)
Item 6	3 (1.9)	35 (22.0)	121 (76.1)
Item 7	1 (0.6)	31 (19.5)	127 (79.9)
Item 8	2 (1.3)	28 (17.6)	129 (81.1)
Item 9	2 (1.3)	36 (22.6)	121 (76.1)
Item 10	1 (0.6)	27 (17.0)	131 (82.4)
Item 11	7 (4.4)	56 (35.2)	96 (60.4)
Item 12	16 (10.1)	52 (32.7)	91 (57.2)
Item 14	1 (0.6)	29 (18.4)	128 (81.0)
Item 15	2 (1.3)	46 (28.9)	111 (69.8)
Item 16	7 (4.4)	41 (25.8)	111 (69.8)
Item 17	7 (4.4)	43 (27.0)	109 (68.6)
Item 18	6 (3.8)	39 (24.5)	114 (71.7)
Item 19	6 (3.8)	30 (18.9)	123 (77.4)
Item 21	4 (2.5)	34 (21.4)	121 (76.1)
Item 22	3 (1.9)	25 (15.7)	131 (82.4)
Item 23	3 (1.9)	32 (20.1)	124 (78.0)
Item 24	4 (2.5)	28 (17.6)	127 (79.9)
Item 25	15 (9.4)	44 (27.7)	100 (62.9)
Item 26	2 (1.3)	41 (25.8)	116 (73.0)
Item 27	0 (0.0)	20 (12.6)	139 (87.4)
Item 28	5 (3.1)	25 (15.7)	129 (81.1)
Item 30	2 (1.3)	27 (17.0)	130 (81.8)
Item 31	3 (1.9)	28 (17.6)	128 (80.5)
Item 33	1 (0.6)	26 (16.4)	132 (83.0)
Item 34	3 (1.9)	23 (14.5)	133 (83.6)
Item 35	1 (0.6)	16 (10.1)	142 (89.3)
Item 36	1 (0.6)	14 (8.8)	144 (90.6)

**Table 5:** Pattern Coefficients for the Three-Factor M-OPSQ Solution Using Direct Oblimin Rotation.

Item	Aesthetics & Psychosocial	Doctor–Patient & Environment	Oral & Dental Function
Item 1	0.537		
Item 2			0.720
Item 3	0.681		
Item 5	0.559		0.309
Item 6	0.521		
Item 7	0.847		
Item 8	0.798		
Item 9	0.829		
Item 10	0.795		
Item 11	0.594		
Item 12	0.399		0.374
Item 14	0.497		
Item 15	0.745		
Item 16			0.930
Item 17			0.931
Item 18			0.865
Item 19		0.320	0.481
Item 21	0.525		
Item 22	0.718		
Item 23	0.302	0.396	
Item 24	0.493	0.329	
Item 25		0.388	
Item 26		0.661	
Item 27		0.687	
Item 28	0.318	0.425	
Item 30		0.653	
Item 31		0.691	
Item 33		0.709	
Item 34		0.821	
Item 35		0.889	
Item 36		0.872	

Note: Pattern coefficients <0.30 are suppressed for clarity. Items were assigned to the factor on which they showed the highest pattern coefficient. Inter-factor correlations were F1–F2 = 0.51, F1–F3 = 0.59 and F2–F3 = 0.35. Item numbers correspond to the original 37-item instrument.

**Table 6:** Comparison of Confirmatory Factor Analysis Model Fit.

Model	χ^2^	df	χ^2^/df	CFI	TLI	RMSEA	SRMR
Unidimensional	2163.9	434	4.99	0.570	0.539	0.159	0.117
Three-factor	1181.0	431	2.74	0.813	0.799	0.105	0.097

**Table 7:** Comparison of Total M-OPSQ Scores by Participant and Treatment Characteristics.

Variable	Median (IQR)	*p*	Test Statistic	Effect Size
Gender				
Male	164.00 (32.75)	0.314 ^a^	U = 2768	r = 0.080 (negligible)
Female	159.50 (26.50)			
Who provided your orthodontic treatment?				
General Dentist	144.00 (28.50)	<0.001 ^b^	H(3) = 16.98	η^2^H = 0.091 (medium)
Orthodontic Specialist	152.00 (27.00)			
Dental Postgraduate Student	167.00 (23.00)			
Unsure/Do Not Know	155.00 (32.50)			
Where did you receive your orthodontic treatment?				
UKM Dental Faculty	165.00 (22.00)	<0.001 ^b^	H(3) = 15.91	η^2^H = 0.084 (medium)
Other University Dental Faculty	153.50 (36.00)			
KKM Dental Clinic	157.00 (31.50)			
Private Dental Clinic	145.00 (24.25)			

^a^ Mann–Whitney U Test. ^b^ Kruskal–Wallis Test. KKM, Kementerian Kesihatan Malaysia (Ministry of Health Malaysia).

**Table 8:** Post hoc Mann–Whitney U Comparisons Between Treatment Provider Groups.

Pairwise Comparison	*p*	Z	r (Magnitude)	Between-Group Difference (95% CI)
General Dentist vs. Orthodontic Specialist	0.107	1.613	0.233 (small)	−13.0 (−24.0 to 3.0)
General Dentist vs. Dental Postgraduate Student	0.003 *	2.971	0.278 (small)	−23.0 (−33.0 to −6.0)
General Dentist vs. Unsure/Do Not Know	0.504	0.668	0.142 (negligible)	−7.0 (−25.0 to 15.0)
Orthodontic Specialist vs. Dental Postgraduate Student	0.008 *	2.637	0.226 (small)	−10.0 (−17.0 to −2.0)
Orthodontic Specialist vs. Unsure/Do Not Know	0.600	0.524	0.079 (negligible)	5.0 (−9.0 to 24.0)
Dental Postgraduate Student vs. Unsure/Do Not Know	0.020	2.319	0.221 (small)	14.0 (5.0 to 31.0)

Bonferroni correction-adjusted alpha: 0.05/6 pairs = 0.0083. * Statistically significant difference.

**Table 9:** Post hoc Mann–Whitney U Comparisons Between Treatment Setting Groups.

Pairwise Comparison	*p*	Z	r (Magnitude)	Between-Group Difference (95% CI)
UKM Dental Faculty vs. Other University Dental Faculty	0.306	1.023	0.091 (negligible)	9.5 (−9.0 to 30.0)
UKM Dental Faculty vs. KKM Dental Clinic	0.949	0.064	0.006 (negligible)	1.0 (−28.0 to 26.0)
UKM Dental Faculty vs. Private Dental Clinic	<0.001 *	3.854	0.318 (small)	18.0 (8.0 to 27.0)
Other University Dental Faculty vs. KKM Dental Clinic	0.360	0.915	0.276 (small)	−9.0 (−41.0 to 19.0)
Other University Dental Faculty vs. Private Dental Clinic	0.439	0.813	0.140 (negligible)	7.5 (−18.0 to 28.0)
KKM Dental Clinic vs. Private Dental Clinic	0.024	2.261	0.394 (small)	15.5 (5.0 to 36.5)

Bonferroni correction-adjusted alpha: 0.05/6 pairs = 0.0083. * Statistically significant difference. KKM, Kementerian Kesihatan Malaysia (Ministry of Health Malaysia).

## Data Availability

The data presented in this study are available on reasonable request from the corresponding author. The data are not publicly available due to ethical restrictions.
